# A Search for Parent-of-Origin Effects on Honey Bee Gene Expression

**DOI:** 10.1534/g3.115.017814

**Published:** 2015-06-05

**Authors:** Sarah D. Kocher, Jennifer M. Tsuruda, Joshua D. Gibson, Christine M. Emore, Miguel E. Arechavaleta-Velasco, David C. Queller, Joan E. Strassmann, Christina M. Grozinger, Michael R. Gribskov, Phillip San Miguel, Rick Westerman, Greg J. Hunt

**Affiliations:** * Department of Organismic and Evolutionary Biology, Harvard University, Cambridge, Massachusetts 02138; † Department of Entomology, Purdue University, West Lafayette, Indiana 47907; ‡ Public Service & Agriculture, Clemson University, Clemson, South Carolina 29634; § Instituto Nacional de Investigaciones Forestales, Agricolas y Pecuarias, Apiculture, 76280 Ajuchitlan, Queretaro, Mexico; ** Department of Biology, Washington University in St. Louis, St. Louis, Missouri 63130; †† Department of Entomology, Center for Pollinator Research, Pennsylvania State University, University Park, Pennsylvania 16802

**Keywords:** imprinting, gene expression, social insects, honey bees, kinship theory, genomics

## Abstract

Parent-specific gene expression (PSGE) is little known outside of mammals and plants. PSGE occurs when the expression level of a gene depends on whether an allele was inherited from the mother or the father. Kin selection theory predicts that there should be extensive PSGE in social insects because social insect parents can gain inclusive fitness benefits by silencing parental alleles in female offspring. We searched for evidence of PSGE in honey bees using transcriptomes from reciprocal crosses between European and Africanized strains. We found 46 transcripts with significant parent-of-origin effects on gene expression, many of which overexpressed the maternal allele. Interestingly, we also found a large proportion of genes showing a bias toward maternal alleles in only one of the reciprocal crosses. These results indicate that PSGE may occur in social insects. The nonreciprocal effects could be largely driven by hybrid incompatibility between these strains. Future work will help to determine if these are indeed parent-of-origin effects that can modulate inclusive fitness benefits.

Parent-specific gene expression occurs when alleles inherited from the mother (matrigenes) and from the father (patrigenes) are expressed at unequal levels in the offspring. Genomic imprinting is a special case of PSGE in which an epigenetic chromosomal mark leads to complete silencing of one of the parental alleles (Haig 2002). The leading evolutionary explanation for PSGE and genomic imprinting is the kinship theory (Haig 2002), which predicts that differences in inclusive fitness between mothers and fathers can lead to conflicts between matrigenes and patrigenes in their offspring. The canonical example concerns an offspring acquiring resources from the mother at the expense of the current or future siblings. The offspring’s patrigenes should favor greater resource acquisition from the mother when some of the siblings that receive fewer resources have a different father.

Kin conflict explains why PSGE is associated with maternal provisioning in mammals and plants, particularly in offspring tissues that influence provisioning (placenta and endosperm). It also explains why patrigene expression tends to enhance offspring growth while matrigenes retard it (Haig 2002). Although studies testing this theory have focused primarily on mother–offspring interactions that occur during development (Babak *et al.* 2008; Dobata and Tsuji 2012), the idea that genomes are divided along lines of parental origin has implications that extend much further. For example, PSGE is predicted to influence cooperative and reproductive behaviors of facultatively nonreproductive workers in social insects (Haig 2002; Queller 2003).

Another, but not mutually exclusive, hypothesis is the mitonuclear coadaptation theory (Wolf 2009). This predicts that interactions between nuclear and mitochondrial genes should favor expression of only the maternal alleles in all lineages. In these circumstances, expression of nuclear genes that interact with mitochondria are predicted to be maternally biased, leading to coadapted expression between nuclear genes and the cytoplasmic organelles that are maternally inherited.

Social insects present a novel system in which to test these predictions (Queller 2003). These organisms spend their lives in colonies interacting with kin that have many matrigene–patrigene differences in relatedness. While PSGE is unknown in these taxa, there are many *a priori* predictions that can be made (Haig 2002; Queller 2003). Honey bees represent one of the most extreme examples of eusociality, where interactions between queen mother and worker daughters are so interwoven that these societies are often referred to as superorganisms. The queen lays eggs that develop either into haploid males or into diploid females that become either sterile workers or reproductive queens. The workers gather, distribute, and defend the nutritive resources of the colony and rear the offspring of the queen. The haplodiploid genetic system and extreme multiple mating by the queen both lead to differential matrigene–patrigene relatedness among workers and to predicted matrigene–patrigene conflict over phenotypes associated with nepotistic rearing of new queens, sex allocation, colony fission, nestmate recognition, and worker reproduction (Haig 2002; Queller 2003; Dobata and Tsuji 2012; Kronauer 2008). DNA methylation is thought to be the primary mechanism mediating PSGE and imprinting; in social ants and bees, the molecular machinery needed for methylation is intact (Bonasio *et al.* 2012; Wang *et al.* 2006; Kocher *et al.* 2013; The Honeybee Genome Sequencing Consortium 2006). Although PSGE in humans and plants usually is due to methylated cytosines in intergenic regions, in social insects methylation occurs primarily within coding regions, and it is often associated with constitutively expressed genes and alternative splicing (Foret *et al.* 2012; Herb *et al.* 2012; Lyko *et al.* 2010; Drewell *et al.* 2014; Hunt *et al.* 2013).

Thus, honey bees appear to have the evolutionary motive (differential matrigene–patrigene relatedness), the means (histone modification and DNA methylation), and the opportunity (interactions with relatives) for PSGE. However, the only direct evidence for parent-specific expression is phenotypic paternal effects on stinging behavior (Guzman-Novoa *et al.* 2005) and worker reproduction (Oldroyd *et al.* 2013), but differential expression of patrigenes and matrigenes have not been examined.

To determine whether PSGE occurs in honey bees, we generated reciprocal crosses by single-drone instrumental insemination between two honey bee lineages: European (*Apis mellifera carnica*) and Africanized (derived from *A. m. scutellata*). These lineages differ at many single nucleotide polymorphisms (SNPs) and for many behavioral and physiological traits, including colony defense, swarming behavior, ovarian activation, and response to queen pheromones (Page and Amdam 2007). The reciprocal cross design uncouples parent-of-origin effects from lineage-of-origin effects that can also impact expression levels (Figure 1).

**Figure 1:**
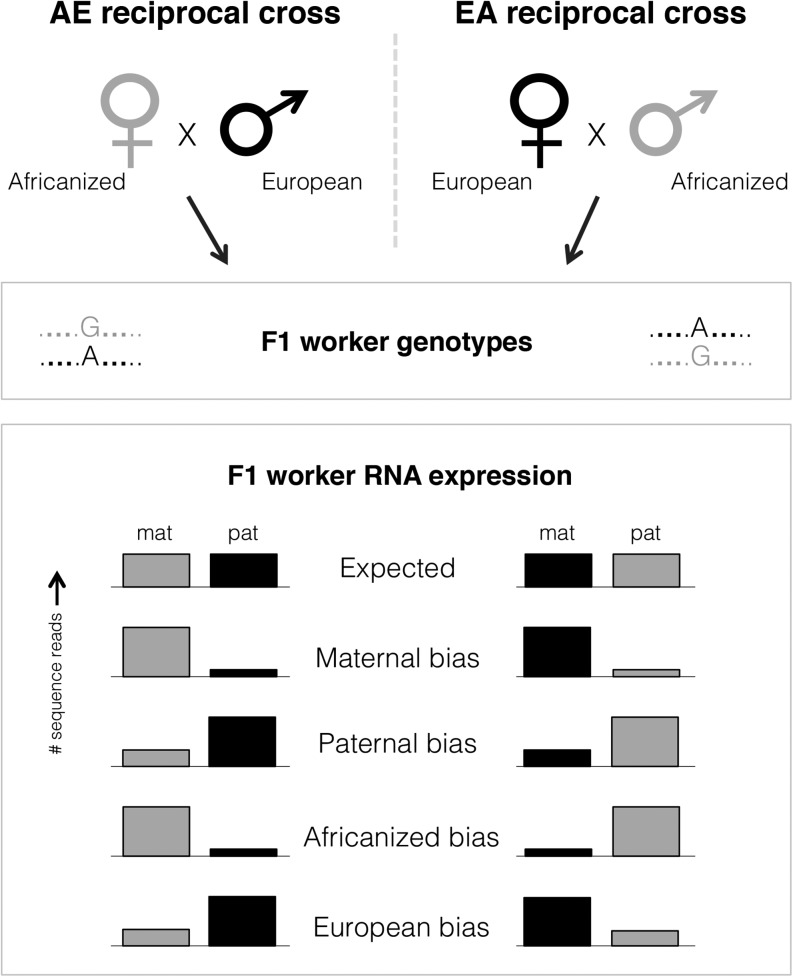
Experimental design. A reciprocal cross design was used to uncouple parent-of-origin effects from lineage-of-origin effects. Two colonies were produced with Africanized maternity (AE) and two colonies with European maternity (EA). As a result, F1 workers from both colonies will be genotypically identical, but the parent-of-origin for each allele varies across colonies. Predicted allelic expression is shown for sites with no bias, a parent-of-origin bias, or a lineage-of-origin (*e.g.*, allelic) bias.

## Materials and Methods

### Summary

To identify SNPs that would enable distinguishing maternal and paternal alleles, we sequenced the genomes of the two queen mothers and drone fathers of the EA (European mother) and AE (European father) reciprocal crosses and mapped the reads to the reference genome (The Honeybee Genome Sequencing Consortium 2006) (Amel4.0). We retained only SNPs where parents were aa × b or bb × a, generating a clear 1 to 1 expectation of read counts in all offspring of each cross (as in Figure 1). We constructed and sequenced worker cDNA libraries consisting of pooled larvae (first instar larvae, EA, n = 2; AE, n = 2), pooled adults (guard bees; EA, n = 2; AE, n = 2), and individual adult brains (foragers; EA, n = 3; AE, n = 3). Reads were mapped to the reference genome, and SNPs in expressed transcripts were then filtered for coverage and sequence quality and analyzed by transcript. A general linear interactive mixed model (SAS, Cary, NC) was implemented for each transcript using counts from maternally and paternally inherited alleles at each SNP to assess parent-of-origin effects on expression. Because analyses were done at the transcript level, this method has the advantage of taking into account variance due to SNP and sample replicates. Parent-of-origin (maternal *vs.* paternal), lineage-of-origin (Africanized *vs.* European), and their interaction were used as fixed terms, and SNP and replicate were included as random factors in the model. We corrected for multiple testing with a false discovery threshold of *P* < 0.05. To generate our list of transcripts showing parent-of-origin biases in gene expression, we required that (a) the parental bias be the same direction in each cross and (b) that the ratio of maternal or paternal reads to the total was greater than 0.6 in both crosses (Wang and Clark 2014). To generate our list of transcripts showing lineage-of-origin biases in gene expression, we required that (a) the lineage bias be the same direction in each cross and (b) that the ratio of Africanized or European reads to the total was greater than 0.6 in both crosses. Finally, to generate our list of transcripts showing a maternal bias in only one of the crosses, we required that transcripts were significant for the interaction term (parent-of-origin*lineage-of-origin) and that the ratio of maternal reads to the total in that cross was greater than 0.6. See Supporting Information, File S1 for additional details.

## Results

### Parent-of-origin effects (PSGE)

**Figure 2:**
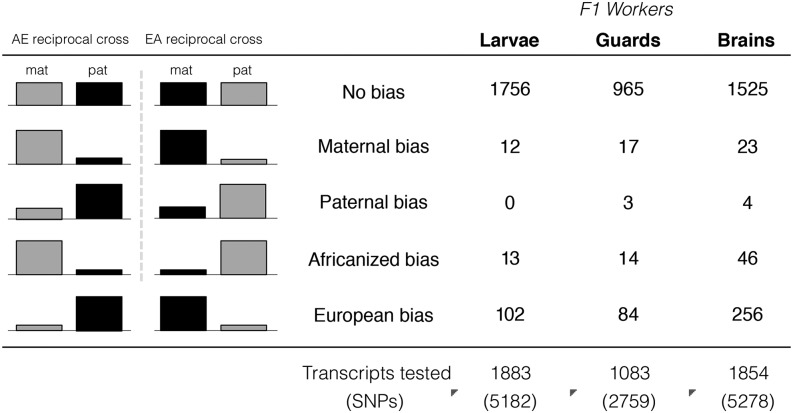
Numbers of transcripts with parentally biased and lineage-biased gene expression. The final row includes the total numbers of tested transcripts and the number of SNPs included in each test are in parentheses. In all cases, there were significantly more maternally biased than paternally biased transcripts (Storer-Kim tests, *P* < 0.001) and more European-biased than Africanized-biased transcripts (Storer-Kim tests, *P* < 0.001). In total, there were 46 transcripts with parent-of-origin effects. Note that some of the transcripts were significant in more than one sampling group (see Table 1).

**Figure 3:**
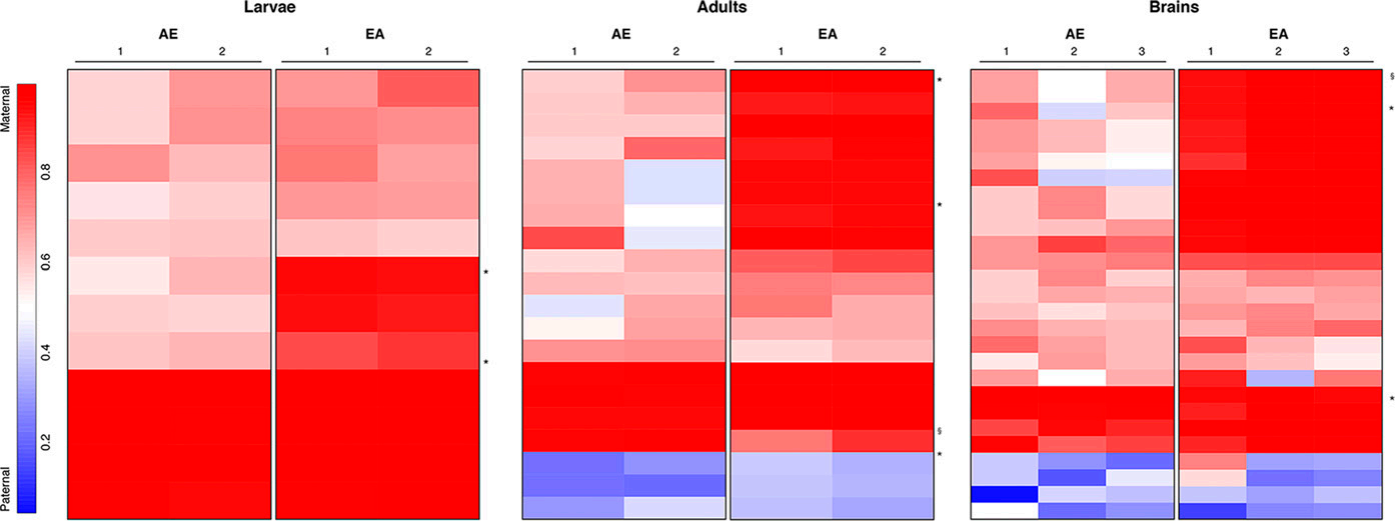
Parent-of-origin effects on gene expression. A heatmap of the 46 parentally biased transcripts demonstrates that reciprocal crosses have somewhat consistent patterns of parental bias across developmental and behavioral states. Each line represents a cufflinks-predicted transcript with a significant parent-of-origin effect on gene expression. Parental bias for each significant transcript is shown for larvae, adults, and brains. AE: cross between Africanized queen and European-derived drone. EA: the reciprocal cross. Numbers under each cross denote replicates. Blues represent a paternal bias; reds represent maternal biases. *The locus was confirmed with validation datasets. ^§^The locus was tested, but the bias was not confirmed. See Table S1 for detailed results.

We identified a total of 46 transcripts with significant parent-of-origin effects on expression (Figure 2, Figure 3, Figure S1, Figure S2) corresponding to 1–2% of the tested transcripts in each sample group (larvae, adults, individual brains). In all sample groups examined, there were significantly more maternally biased than paternally biased transcripts (Figure 2) (Storer-Kim tests, *P* < 0.001). These transcripts are distributed throughout the genome and do not appear to form any distinct clusters.

To validate our results, we used a combination of pyrosequencing and Illumina MiSeq (Wittkopp 2011; Deveale *et al.* 2012). Five of seven tested transcripts were confirmed using these methods (Figure 3, Table S1). There was no significant association between parental bias and known methylated transcripts (Herb *et al.* 2012; Lyko *et al.* 2010; Elango *et al.* 2009; Foret *et al.* 2012) (hypergeometric test, *P* > 0.05 in all instances). These results are not necessarily surprising because, in contrast to plants and mammals, methylation occurs primarily in the gene bodies in Hymenopteran species and is often targeted to constitutively expressed genes (Hunt *et al.* 2013). Therefore, it is entirely possible that PSGE may be modulated by an entirely different mechanism in this group.

The overlap in the set of biased transcripts among the three different sample groups (larvae, adults, and brains) was significantly more than expected by chance (Table 1) (hypergeometric tests, *P* < 0.05). Two transcripts were common across all three groups: XLOC_012772 and XLOC_013553. Both overexpressed the maternal allele, and both are copies of mitochondrial oxidoreductase sequences transposed into the nuclear genome (Behura 2007) (BLASTn; e-value < 10^−3^). Approximately 2% of the nucleotides in XLOC_012772 have diverged from the mitochondrial COII sequence, whereas more than half of the nucleotides in XLOC_013553 match the mitochondrial COI sequence. It is unlikely this result was a byproduct of the mapping of mitochondrial reads to the nuclear genome because only uniquely mapped reads were considered for downstream analyses and the mitochondrial sequence was present in the reference genome.

**Table 1:** Significant overlap among gene lists was assessed using a hypergeometric test

	Larvae	Adults	Brains
Larvae	—	3*^a^*	3*^a^*
		*(872)*	*(1182)*
Adults	—	—	4*^b^*
			*(750)*
Brains	—	—	4*^b^*
			*(750)*

Italicized numbers indicate the number of tested transcripts that were shared between tissues. There was substantially more overlap between all three lists than expected by chance (hypergeometric test, *P* < 0.05).

a *P* < 0.005.

b *P* < 0.05.

In general, maternally biased transcripts in both crosses (see Table S1 for a complete list) were associated with transcriptional function, including two genes whose *Drosophila* homologs have been associated with chromatin remodeling and regulation of gene expression: *brahma* (GB13881) (Armstrong *et al.* 2002) and *trithorax-related* (GB41196) (Srinivasan *et al.* 2005). Several other genes were associated with neural function and development, including *aurora* and *bifocal*, both of which are associated with the regulation of neurogenesis (Neumüller *et al.* 2011; Babu *et al.* 2005), and *Tenascin accessory* (*ten-A*; GB42976), which is associated with synaptic growth and helps to establish proper connectivity in the mushroom bodies and central complex of the brain (Hong *et al.* 2012). The majority of paternally overexpressed transcripts are uncharacterized in *Drosophila*, but two of these transcripts have some similarity to members of the opsin gene family, and one of these transcripts is homologous to *pirk* (GB44455), a gene known to negatively regulate immune response (Kleino *et al.* 2008).

#### No evidence for mito-nuclear coadaptation:

The mitonuclear coadaptation theory predicts the strong matrigene-biased expression in both reciprocal crosses if the PSGE transcripts are involved in interactions with the mitochondria (Wolf 2009). We used reciprocal BLASTs to compare our list of 46 transcripts to 690 protein-coding genes that localize to mitochondria in *Drosophila melanogaster* (Smith *et al.* 2012; Pagliarini *et al.* 2008). Of the 46 transcripts, only two were on this list: one was maternally biased (GB49079) and the other was paternally biased (GB46444). Thus, we found no evidence for coadaptation theory in our dataset (chi-square, *P* = 0.95). However, it is important to note that this was a conservative test using only reciprocal best matches to protein coding genes.

#### Lineage-of-origin effects:

There were 442 transcripts with a significant lineage-of-origin effect on gene expression (Figure 2, Table S2). Most of these transcripts (n = 378) were biased toward overexpression of European rather than Africanized alleles. This bias in expression is highly robust and consistent across different tissues and developmental stages, and simulations demonstrate that these results are not due to ascertainment bias alone (Table S4; see File S1 for details).

Overall, transcripts with a significant lineage-of-origin effect on gene expression were associated with chitin binding (*P* = 0.003) and carbohydrate binding (*P* = 0.003) (Dennis *et al.* 2003). Notably, eight of these transcripts are located within the *Pln-1* locus (XLOC_000365, XLOC_000384, XLOC_000407, XLOC_000410, XLOC_000828, XLOC_000850, XLOC_003272, and XLOC_003327), which has been linked to the regulation of foraging behavior in honey bees (Page and Amdam 2007).

#### The interaction of parental and lineage effects:

In many instances, we found evidence for a significant interaction of parent-of-origin and lineage-of-origin on gene expression. For example, of the 46 parentally biased transcripts, 30 were also significantly associated with the interaction term in our model (FDR < 0.05), demonstrating that the magnitude of the parent-of-origin effects often varied depending on the lineage-of-origin. Twenty-seven of these showed greater overexpression of the EHB maternal allele relative to AHB maternal alleles (*i.e.*, when the maternal allele was European, there was often a much stronger parent-of-origin effect).

Among all of the transcripts significant for the interaction term in our model, a strong bias was often observed toward overexpression of the maternal allele in hybrids with European maternity (the EA family) rather than hybrids with Africanized maternity (AE). Of these, we found 319 transcripts with a significant maternal bias in one of the crosses, 83% of which were maternally biased only in the EA family (Table S3). We found a strikingly similar pattern in the validation gene set, where five of seven tested transcripts that were more maternally biased in one of the reciprocal crosses were similarly biased in the validation (Table S3).

#### The potential role of mito-nuclear incompatibilities:

The mito-nuclear coadaptation theory predicts that maternal biased expression should occur in both hybrid families due to coadaptation in both parental lineages (Wolf 2009). However, mito-nuclear incompatibilities may occur in hybrids due to the breaking apart of coadapted complexes, and these incompatibilities are expected to be manifested asymmetrically in one of the two hybrid families (Turelli and Moyle 2007). To determine whether increased expression of matrigenic alleles in one family might be a result of mito-nuclear incompatibilities between Africanized and European alleles in honey bees, we tested for over-representation of genes known to express proteins translocated to the mitochondria (Smith *et al.* 2012; Pagliarini *et al.* 2008) among all 319 transcripts maternally biased in at least one cross. We found significantly more of these genes than expected by chance in the list of transcripts that were maternally biased in the EA larvae (hypergeometric test, *P* = 0.0015), suggesting that this may indeed be the case. Thus, the maternal biases found in many of the significant transcripts in this study may also be explained by mito-nuclear incompatibilities between Africanized and European honey bee strains; therefore, we cannot rule this out as a major effect on gene expression.

## Discussion

We have documented a set of 46 transcripts showing significant parent-of-origin effects on gene expression. These results are consistent with evolutionary theory predicting that PSGE should occur in social insect species (Haig 2002; Queller 2003). Our data show more extreme PSGE when the matrigene is overexpressed than when the patrigene is overexpressed. This could be explained by the fact that matrigenes are also passed to haploid sons, where strongly reducing expression may be harmful unless there are mechanisms preventing the change of expression in sons only.

Our results suggest that PSGE in honey bees may be a continuous gradient of expression levels rather than all-or-none. It is possible that these results are an artifact of our experimental design because whole bodies of multiple individuals were pooled for the larval and adult samples. However, we also examined brains of individual bees and found similar results, suggesting that neither tissue-specific effects nor pooling are likely to drive the observed patterns. Alternatively, it could be that hybrid incompatibilities between the strains of bees used in this study destabilize PSGE and lead to an overall increase in biallelic expression in the offspring (Wolf *et al.* 2014). Often, hybrid incompatibilities lead to aberrant gene expression patterns and a loss of imprinting in one direction of the cross (Wolf *et al.* 2014), and our data show a similar pattern. Thus, it is possible that PSGE occurs more extensively throughout the honey bee genome, but that incompatibilities between the two strains used in this study masked additional PSGE. Future work using reciprocal crosses from the same subspecies is needed to convincingly demonstrate the extent of PSGE within honey bees.

Two further aspects of these results raise puzzles for future work. First, we found no association between parental bias and known methylated transcripts (Herb *et al.* 2012; Lyko *et al.* 2010; Elango *et al.* 2009; Foret *et al.* 2012). The mechanisms underlying parentally biased gene expression in insects are largely unknown: although methylation in mammals appears to be strongly correlated with gene expression, methylation in insects is much more strongly linked to alternative splicing and is targeted to constitutively expressed genes (Flores *et al.* 2012; Foret *et al.* 2012; Park *et al.* 2011; Hunt *et al.* 2013). To better test these associations, however, more studies are needed examining methylation, histone modifications, and allele-specific expression in the same sample set. Second, we identified one gene that is maternally biased in both families that is located within a QTL associated with stinging behavior, a phenotype for which *paternal* effects have been documented (Guzman-Novoa *et al.* 2005). This transcript corresponds to the gene, *huntingtin*, a known candidate gene for stinging behavior that modulates neuronal transcription and that plays a role in synaptic transmission, intracellular neuronal transport, and dendrite morphology (Guzman-Novoa *et al.* 2005; Hunt *et al.* 2007; Harjes and Wanker 2003; Li and Li 2004). There are many scenarios in which this pattern might make sense. For example, paternal silencing (or partial silencing) of the patrigene could reduce overall gene expression levels, thereby modulating aggression in workers. Alternatively, it could be that *huntingtin* has indirect effects (*e.g.*, trans-regulation) on other genes that impact aggression.

The kin conflict theory predicts an excess of imprinted genes related to worker social behaviors and reproduction. There seems to be some evidence supporting this at the phenotypic level, where parent-of-origin effects have been described for worker reproduction (Oldroyd *et al.* 2013). To answer this question directly, these effects would also need to be studied at the transcriptional level. Although our dataset did not examine reproductive workers, we did find some interesting maternally biased transcripts. One of these was *Neural Lazarillo* (*AmNLaz*; GB50875), which downregulates insulin signaling, decreasing growth and increasing lipid storage in both humans and flies (Page and Amdam 2007; Pasco and Léopold 2012). Insulin signaling is a major pathway underlying queen–worker caste differentiation in honey bees, and downregulation of nutrient sensing genes in this pathway leads to a worker-like phenotype (Wheeler *et al.* 2006; Mutti *et al.* 2011; Mullen *et al.* 2014; Patel *et al.* 2007). Kinship theory would predict maternal expression of such a protein (Queller 2003) and, indeed, we found nearly complete expression of the maternal alleles in honey bee larvae (Figure 1), lending support to these theoretical predictions.

Another major pattern in our data was the asymmetrical expression bias of alleles within the cross with European maternity in a large number of transcripts. This same pattern is observed for phenotypic traits at the population level within the invasive hybrid Africanized bees (Hall and Muralidharan 1989), and we documented 319 transcripts showing this interaction effect, 287 of which were biased toward expression of the European maternal allele. The restriction of maternal expression bias for many genes to one reciprocal family is novel, and the mechanism underlying this phenomenon is currently unknown. However, this pattern matches the broad pattern of asymmetric dysfunction in hybrid families (Turelli and Moyle 2007) and also matches the asymmetric pattern of metabolic deficits in hybrids with European maternity found in crosses between these honey bee races (Harrison and Hall 1993). Given that mitochondria are responsible for producing the majority of cellular energy through aerobic metabolism, the similarity of these patterns suggests that nuclear–mitochondrial incompatibility may be occurring in these hybrids, and that expression of the matrigenic alleles may be a means to modulate these incompatibilities (Wolf 2009). In the larvae, and only in the EA family, the asymmetrically biased genes are significantly enriched for genes that localize to mitochondria, suggesting that nuclear–mitochondrial interactions may indeed underlie this asymmetry. This pattern also reflects the asymmetric pattern in aggressive behavior, which is higher in EA hybrids and has been linked to reduced brain metabolic rates (Guzman-Novoa *et al.* 2005; Li-Byarlay *et al.* 2014; Alaux *et al.* 2009).

## Conclusions

In summary, we have found some evidence for parent-of-origin effects on gene expression in honey bees, although the results of this study are not conclusive. If there is PSGE in the social insects, then the mechanism may not be the same as PSGE in other taxa. The majority of the parentally biased transcripts we detected overexpressed the maternal allele. Importantly, we also found a large set of genes with an asymmetric bias toward the maternal alleles in only one of the crosses, suggesting that nuclear–mitochondrial interactions may be playing a role in European and Africanized honey bee hybridization. Although these results do not clearly demonstrate PSGE in honey bees, they do confirm that the social insects will provide fertile ground for testing evolutionary theories related to PSGE and genomic imprinting. These exemplars of cooperation among individuals will be excellent models for studying conflict within individual genomes (Haig 2002). Paradoxically, cooperation *between* individuals and conflict *within* individuals may be linked through parent-of-origin effects on gene expression.

## Supplementary Material

Supporting Information
