# Supplementary material for: A Search for Parent-of-Origin Effects on Honey Bee Gene Expression
Source: G3 (Bethesda). 2015 Jun 5;5(8):1657–62. doi: 10.1534/g3.115.017814 (PMC4528322; doi:10.1534/g3.115.017814)
Supplement: Supporting Information [file supp_5_8_1657__index.html]

A Search for Parent-of-Origin Effects on Honey Bee Gene Expression — Supporting Information 

# A Search for Parent-of-Origin Effects on Honey Bee Gene Expression

## Supporting Information for Kocher *et al.*, 2015

**Files in this Data Supplement:**

- Supporting Information - File S1 and Tables S1-S5 (PDF, 212 KB)
- File S1 - Supplemental Materials and Methods (PDF, 189 KB)
- Table S5 - Depth of sequencing coverage for all samples, related to Experimental Procedures. (PDF, 142 KB)
- Table S1 - Parentally-biased transcripts, related to Experimental Results. Transcripts with a significant parent-of-origin effect on gene expression. The first column includes the annotations based on a BLASTp search. XLOC ID indicates the annotated transcript ID as output by Cufflinks. Data values indicate the proportion of maternal (or paternal) reads relative to the total number of reads for each sample replicate. Data are presented the same way for both the pyrosequencing and MiSeq validation methods. (.xlsx, 31 KB)
- Table S2 - Lineage-biased transcripts, related to Experimental Results. Transcripts with a significant lineage-of-origin effect on gene expression in both crosses. As previously, the first column includes the annotations based on a BLASTp search. XLOC ID indicates the annotated transcript ID as output by Cufflinks. Data values indicate the proportion of Africanized (or European) reads relative to the total number of reads for each sample replicate. (.xlsx, 149 KB)
- Table S3 - Maternally-biased transcripts, related to Experimental Results. Transcripts with a significant maternal effect on gene expression in one cross. As previously, the first column includes the annotations based on a BLASTp search. XLOC ID indicates the annotated transcript ID as output by Cufflinks. Data values indicate the proportion of maternal (or paternal) reads relative to the total number of reads for each sample replicate. Data are presented the same way for both the pyrosequencing and MiSeq validation methods. (.xlsx, 127 KB)
- Table S4 - Ascertainment bias, related to Experimental Results. Transcripts predicted to show significant maternal biases in the EA cross due to ascertainment bias mapping effects. Columns as described above. (.xlsx, 51 KB)
