# Supplementary material for: A Search for Parent-of-Origin Effects on Honey Bee Gene Expression
Source: G3 (Bethesda). 2015 Jun 5;5(8):1657–62. doi: 10.1534/g3.115.017814 (PMC4528322; doi:10.1534/g3.115.017814)
Supplement: Supporting Information [file supp_g3.115.017814_FileS1.pdf]

File S1

Supplemental Materials and Methods.

Crosses. Five Africanized honey bee (AHB) colonies and four European honey bee (EHB; *A.m. carnica*) colonies were maintained in at INIFAP facilities in Tonatico, Estado de México, Mexico. Colonies were identified as Africanized based on mitochondrial haplotype and morphometrics. The EHB lineages had been maintained by instrumental insemination. The estimated F_{ST} between *A.m. ligustica* (a close relative to *A.m. carnica*) and Africanized strains ranges from 0.385-0.406 (Whitfield *et al.* 2006).One AHB and one EHB colony were chosen based on extreme differences in stinging response (Shorter *et al.* 2012) and attraction to queen mandibular pheromone; AHB are less responsive to this pheromone (Kocher, Hunt, and Grozinger, *unpublished data*).

Sample handling and sequencing. Potential biases in measurement of allele specific expression caused by low library complexity were controlled by using multiple libraries per sample and multiple samples. RNA was extracted from pools of larvae (n= 90 individual first instar larvae in pool 1, n=75 in pool 2) and pools of adults (n = 56 individual guards per pool) with Trizol (Invitrogen, Grand Island, NY). Brains of individual adults were dissected on dry ice and RNA was extracted with the PicoPure kit (Life Technologies, Grand Island, NY) and amplified. To identify SNPs that would enable distinguishing maternal and paternal alleles, we extracted DNA from the two queen mothers and drone fathers of the EA (European mother) and AE (European father) reciprocal crosses using the DNeasy Blood and Tissue kit from Qiagen (Valencia, CA), sequenced their genomes and mapped the reads to the reference genome (Amel4.0 (Consortium 2006); Table S4). Both drone genomes were sequenced on an ABI SOLiD, and queen genomes were sequenced using a combination of data from the ABI SOLiD as well as an Illumina HiSeq.

To assess parent-specific biases in gene expression, we sequenced the RNA of pooled larvae and pooled adults; two libraries of each sample group were constructed for both the AE and EA reciprocal colonies. We also sequenced the brains of 3 individual adults from each reciprocal colony (AE and EA). RNAseq libraries were sequenced to high coverage on an Illumina HiSeq platform (~200x per sample; Table S4).

Read processing and SNP calling for parental genomes. Sequencing reads were first processed in FastX v.0.0.13 (*http://hannonlab.cshl.edu/fastx_toolkit/index.html*) to examine data quality; read quality was also visually inspected in IGV (Robinson *et al.* 2011). Reads were trimmed when the quality decreased below 20. Trimmed genomic DNA reads were then

input into Novoalign (http://www.novocraft.com) for alignment to the *Apis mellifera* 4.0 genome assembly(Consortium 2006) (www.hgsc.bcm.tmc.edu/project-species-i-Apismellifera.hgsc?pageLocation=Apis mellifera) with the following options: -k, -H, -Q 10. Briefly, these options required that reads had a minimum alignment quality of 10, with quality calibration and hard clipping of low quality bases on the 3' end both enabled. Detailed descriptions of these options can be found in the Novoalign manual (http://www.novocraft.com). Reads that aligned to multiple regions were excluded from subsequent analyses.

Bam files were processed in Picard Tools v1.62. SNP calling was according to the Best Practice Variant Detection protocol available for the Genome Analysis Toolkit v2.0.39(McKenna *et al.* 2010). SNPs were called using the Unified Genotyper, and were also called using samtools mpileup (Li *et al.* 2009). Variants with quality scores < 30 and/or mapping qualities < 40 were excluded from downstream analysis. After variant filtration, the Unified Genotyper and mpileup showed a high degree of concordance. The SNPs generated from the Unified Genotyper were used for subsequent analyses.

There were 2,896,401 SNPs that varied between queens and passed quality control. Of these, 2,340,162 were heterozygous sites, and 556,239 were homozygous in both queens. The drone alleles matched the queen alleles of the same race at 547,964 SNPs, yielding the desired aa x b and bb x a combination. 264,597 of these SNPs were located within expressed regions. SNPs were confirmed by sequencing the genomic DNA from 44 pooled workers from the AE F1 colony. 53,364 were called by the Unified Genotyper as either homozygous at that site (n=53,181) or heterozygous with different allelic calls (n=246); these sites were excluded from further analyses. Genotype calls were further confirmed with the pooled F1 cDNA. If the parental genotypes and F1 genotypic data were inconsistent, this site was excluded from subsequent analyses. After filtering, 203,637 overlapped with expressed regions in the F1 RNA-seq datasets, and were used for allele-specific expression analyses in SAS.

Read processing and SNP calling for offspring transcriptomes. cDNA reads were also visually inspected and processed as previously described using FastX prior to downstream processing. These reads were then aligned using Tophat v2.0.6, and transcript predictions were produced using Cufflinks v1.1.0. Transcripts were assembled using Cuffmerge 2.0.2 using the *Apis mellifera* official gene set version 1.0 with manual annotations (OGS1.0) (Trapnell *et al.* 2012).

The outermost boundaries for each transcript were established by using the coordinates of the longest isoform associated with each transcript that did not overlap the next adjacent transcript. In total, there were 203,637 SNPs that were expressed in the

F1 worker transcriptomes. For inclusion in downstream analyses, a total of least 8 reads per site was required for each replicate in each sample.

After filtering for quality and coverage, there remained 1,883 unique transcripts containing 5,182 SNPs in the larvae, 1,083 transcripts containing 2,759 SNPs in the adults, and 1,854 transcripts containing 5,278 SNPs in the brains. Mean coverage per SNP was calculated for each replicate. Larvae had a mean per-SNP coverage of 91.75 (90.66 in AE colony, and 92.86 in EA), the adult guards had a mean per-SNP coverage of 213.24 (88.40 in AE; 338.19 in EA), and the adult brains had a mean per-SNP coverage of 146.32 (130.0 in AE; 162.7 in EA). To get the count data from the F1 cDNA, pileups were generated for both parents and F1s using the pileup function in Samtools v0.1.12a. Read counts were then parsed from the pileup file using a custom C++ script, and then imported into SAS v9.2 (Cary, NC) for subsequent statistical analyses.

Statistics. A general linear interactive mixed model (GLIMMIX) was implemented for each transcript using counts from each allele at each SNP to assess parent-of-origin effects on expression. Because analyses were done at the transcript-level, this method has the advantage of taking into account variance due to SNP and sample replicates. Parent-of-origin (maternal vs. paternal), race-of-origin (AHB vs. EHB), and their interaction (parent*race) were used as fixed terms, and SNP and replicate were included as random factors in the model. We corrected for multiple testing with a false discovery threshold of $p<0.05$. Notably, though the reciprocal design attempts to control for allele-specific effects, we still detected an overall bias towards the reference alleles, with most maternally biased transcripts showing stronger bias in the EA cross (European mother). This could result from poorer mapping of some diverged African sequences, and therefore we validated our methods using library and mapping independent methods.

Ascertainment bias. To ensure that the observed patterns were not due to ascertainment bias, we mapped genomic reads (with a clear expectation of a 1:1 allelic ratio) from an independent set of individuals from the AE cross to the reference genome and found a 1:1 allele ratio, demonstrating that ascertainment bias could not explain the asymmetry. We calculated the average ratio of AE to EA for SNP loci with coverage in the 20 to 40x range and found that the ratio was 50.2%. Excluding SNPs with flanking indels, a chi square test was performed for each of the remaining loci. 37 SNPs in 30 loci failed the chi square test at a 0.05 level, suggesting that there may be a mapping bias for approximately 30 loci (Table S3). However, all but four of these loci contained an equal or greater number of SNPs that passed the chi square test, suggesting that these effects may be isolated to individual SNPs. Only 13 of these loci overlapped with our significant genes showing maternal biases (n=317

significant transcripts), allowing us to rule out the possibility of ascertainment bias in generating the overall bias towards overexpression of the maternal alleles (hypergeometric test, p=0.68).

We also minimized the possibility that these effects were caused by artifacts of library construction because we used seven different libraries for each family and our results were validated by methods independent of potential library construction bias. We amplified selected transcripts from these samples as well as independent samples and sequenced them on an Illumina MiSeq platform. A second validation was performed by pyrosequencing to provide an additional test that involves neither read mapping nor library construction (see below).

Validation of parent-of-origin effects. We performed two independent validations, using library-independent MiSeq sequencing of amplicons and library- and mapping-independent pyrosequencing.

(1) MiSeq. First, validation was performed within each reciprocal cross using library-independent sequencing of amplicons on the Illumina MiSeq platform. Sequencing amplicons provided an independent method that did not involve biases that may arise in HiSeq library construction. RNA from pools of 75 larvae (all of which were in the original HiSeq pools), 60 whole-body adult guards (most of which were in the original pools), and a different set of 40 whole-body adult foragers from the same colonies (but not represented in the HiSeq sequencing run) was prepped according to TruSeq sample preparation methodologies (Illumina, San Diego, CA). PCR primers were designed using Primer3(Untergasser *et al.* 2012) and Amplify3 (http://engels.genetics.wisc.edu/amplify/) software to produce amplicons containing 1-3 SNPs of interest from transcripts across the genome. Amplicons ranged from 70-350 bp and paired-end sequencing resulted in 2 x 150 bp reads. Reads were mapped to the target sequences using Burrows-Wheeler Aligner (BWA), discarding reads with quality scores <20. SNPs were visually inspected using IGV(Robinson *et al.* 2011) and read counts were quantified. Chi-square tests were used to evaluate significant deviation from a 1:1 ratio of maternal to paternal read counts (as is expected when no bias is present) within each reciprocal cross (EA and AE). Validation was confirmed when expression patterns of the amplicon data matched the expression pattern of the HiSeq transcript data.

(2) Pyrosequencing. Validation with pyrosequencing was performed to ensure that mapping bias did not skew our results. Primers for pyrosequencing were designed for transcripts showing maternal expression bias in the EA cross using Pyromark Assay Design 2.0 (Qiagen, Valencia, CA) and the highest scores for each transcript were chosen for primer synthesis. RNA

sample pools were the same as utilized for the MiSeq validation. cDNA was generated from the pools of RNA from larvae and adults as well as from individual brains using an Oligo $(dT)_{18}$ primer and the Tetro cDNA Synthesis Kit (Bioline USA Inc, Taunton, MA). PCR was performed according to the manufacturer's instructions using the PyroMark PCR Kit (Qiagen, Valencia, CA). For each SNP to be analyzed, DNA of the queens from each reciprocal family was sequenced alongside the cDNA of their daughters in order to ensure that the queens were homozygous and different at that SNP, thereby ensuring pools of heterozygous daughters. Pyrosequencing was performed at the genomics core facility at the Fred Hutchinson Cancer Research Center (Seattle, WA) on a PyroMark Q96 MD (Qiagen). We were unable to perform a goodness of fit test due to the nature of the PyroMark data (proportions of alleles rather than counts). We therefore simulated data sets with varying total read counts to determine a threshold level of parental bias that would be significant. We used total counts of 50, 75 and 100 reads to determine if the increase in assumed read count would bias our results by lowering the parental bias threshold. We used these values because all but two of our transcripts to be validated had >75 total reads in our RNAseq data set. Using these total read counts, we calculated the minimum parental bias necessary to achieve significance at our conservative Bonferroni corrected alpha value (bias = +/-0.48; $p < 0.001$ for 48 total comparisons). We considered transcripts to be validated if they were significantly biased in the same direction in both the HiSeq and PyroMark datasets.

Manual annotation of transcripts*.* Predicted proteins (based on the OGS3.21.1(Elsik *et al.* 2014) gene set) were used in BLASTp searches of NCBI's non-redundant databases. Homologous proteins were evaluated for function in literature searches. Transcripts with no predicted gene model were used in BLASTx searches and analyzed for open reading frames to identify putative ncRNA. Putative *Drosophila* orthologs were determined by reciprocal BLASTp between OGS3.2 and *Drosophila* peptide sequences and a minimum score of $1e^{-10}$. Annotations are shown in Additional files 1 and 2.

In order to determine if our biased gene set is enriched for genes that localize to the mitochondria, we performed a reciprocal BLASTp between the honey bee official gene set (OGS 3.2) proteins(Elsik *et al.* 2014) and a set of *Drosophila melanogaster* orthologs of nuclear-encoded mouse genes that have been experimentally and computationally determined to localize to mitochondria; the mouse Mitocarta gene set 16 (*D. melanogaster* orthologs(Smith *et al.* 2012); mouse Mitocarta(Pagliarini *et al.* 2008)). We performed a BLAST of the Mitocarta genes against the honey bee OGS3.2 and a reciprocal BLAST of OGS3.2 against the Mitocarta genes.

The single best matching sequence to each query was selected based on the e-value, followed by percent identity (if e-values were identical). Only reciprocal best hits were included in downstream analyses (i.e. fly Mitocarta gene *1* best matches honey bee gene *A* in the first BLAST, and honey bee gene *A* best matches mouse Mitocarta gene *1* in the second BLAST). This ensures that the two genes are most likely orthologous. We then used the count of orthologous genes (those that localize to mitochondria) relative to the total number of genes in the honey bee OGS3.2 as a null hypothesis to test whether there is an overrepresentation of these genes in our biased gene set after removing genes that don't code for proteins. We tested this using an exact hypergeometric test.

Gene ontology and overlap with published studies*.* The significantly-biased transcripts from each sample group were compared to several published methylation datasets(Foret *et al.* 2012; Herb *et al.* 2012; Lyko *et al.* 2010; Elango *et al.* 2009). The number of overlapping genes was evaluated using a two-tailed hypergeometric test to determine if there were significantly more or less genes represented on both lists than expected by chance. Gene ontology analyses were conducted using DAVID(Dennis *et al.* 2003). Orthologous genes were determined using the BLASTp algorithm to identify the best *D. melanogaster* match to the predicted honey bee genes.

Supplementary References

Consortium, H. G. S., 2006 Insights into social insects from the genome of the honeybee, Apis mellifera. Nature 931–939.

Dennis, G. J., B. T. Sherman, D. A. Hosack, J. Yang, W. Gao *et al.*, 2003 DAVID: Database for Annotation, Visualization, and Integrated Discovery. Genome Biology 4: P3.

Elango, N., B. G. Hunt, M. A. D. Goodisman, and S. V. Yi, 2009 DNA methylation is widespread and associated with differential gene expression in castes of the honeybee, Apis mellifera. Proceedings of the National Academy of Sciences 106: 11206–11211.

Elsik, C. G., K. C. Worley, A. K. Bennett, M. Beye, F. Camara *et al.*, 2014 Finding the missing honey bee genes: lessons learned from a genome upgrade. BMC genomics 15: 86.

Foret, S., R. Kucharski, M. Pellegrini, S. Feng, S. E. Jacobsen *et al.*, 2012 DNA methylation dynamics, metabolic fluxes, gene splicing, and alternative phenotypes in honey bees. Proceedings of the National Academy of Sciences 109: 4968–4973.

Herb, B. R., F. Wolschin, K. D. Hansen, M. J. Aryee, B. Langmead *et al.*, 2012 Reversible switching between epigenetic states in honeybee behavioral subcastes. Nature Neuroscience 15: 1371–1373.

Li, H., B. Handsaker, A. Wysoker, T. Fennell, J. Ruan *et al.*, 2009 The Sequence Alignment/Map format and SAMtools. Bioinformatics 25: 2078–2079.

Lyko, F., S. Foret, R. Kucharski, S. Wolf, C. Falckenhayn *et al.*, 2010 The honey bee epigenomes: differential methylation of brain DNA in queens and workers. PLoS Biology 8: e1000506.

McKenna, A., M. Hanna, E. Banks, A. Sivachenko, K. Cibulskis *et al.*, 2010 The Genome Analysis Toolkit: A MapReduce

framework for analyzing next-generation DNA sequencing data. Genome Research 20: 1297–1303.

Pagliarini, D. J., S. E. Calvo, B. Chang, S. A. Sheth, S. B. Vafai *et al.*, 2008 A Mitochondrial Protein Compendium Elucidates Complex I Disease Biology. Cell 134: 112–123.

Robinson, J. T., H. Thorvaldsdóttir, W. Winckler, M. Guttman, E. S. Lander *et al.*, 2011 Integrative genomics viewer. Nature Biotechnology 29: 24–26.

Shorter, J. R., M. Arechavaleta-Velasco, C. Robles-Rios, and G. J. Hunt, 2012 A Genetic Analysis of the Stinging and Guarding Behaviors of the Honey Bee. Behav Genet 42: 663–674–674.

Smith, A. C., J. A. Blackshaw, and A. J. Robinson, 2012 MitoMiner: a data warehouse for mitochondrial proteomics data. Nucleic Acids Research 40: D1160–D1167.

Trapnell, C., A. Roberts, L. Goff, G. Pertea, D. Kim *et al.*, 2012 Differential gene and transcript expression analysis of RNA-seq experiments with TopHat and Cufflinks. Nature protocols 7: 562–578.

Untergasser, A., I. Cutcutache, T. Koressaar, J. Ye, B. C. Faircloth *et al.*, 2012 Primer3--new capabilities and interfaces. Nucleic Acids Research 40: e115–e115.

Whitfield, C. W., S. K. Behura, S. H. Berlocher, A. G. Clark, J. S. Johnston *et al.*, 2006 Thrice Out of Africa: Ancient and Recent Expansions of the Honey Bee, Apis mellifera. Science 314: 642–645.
