# Supplementary material for: A Search for Parent-of-Origin Effects on Honey Bee Gene Expression
Source: G3 (Bethesda). 2015 Jun 5;5(8):1657–62. doi: 10.1534/g3.115.017814 (PMC4528322; doi:10.1534/g3.115.017814)
Supplement: Supporting Information [file supp_g3.115.017814_TableS5.pdf]

Table S5. Depth of sequencing coverage for all samples, related to Experimental Procedures.

The number of mapped reads and properly paired reads for each sample replicate. Mean coverage is calculated as (# mapped reads*read length(bp)) / (total sequence length(bp)).

Sample	Total reads	Mapped	Paired	Coverage*
6R_drone	117142832	74702857	64391032	**29.38**
8B_drone	124991604	85535921	73635406	**33.64**
6R_queen	114949298	97323965	92094406	**41.79**
8B_queen	391327620	347122687	332117964	**149.05**
Instars_A_Blanco	30229044	30229044	26885324	**167.53**
Instars_B_Blanco	35767847	35767847	31858708	**198.22**
Instars_A_Rojo	33813595	33813595	29820526	**187.39**
Instars_B_Rojo	32879078	32879078	29219912	**182.21**
Guards_1_Blanco	37275881	37275881	32898690	**206.58**
Guards_2_Blanco	44503015	44503015	38681402	**246.63**
Guards_1_Rojo	12310104	12310104	10789138	**68.22**
Guards_2_Rojo	10702645	10702645	9365404	**59.31**
Brains_1_Blanco	68461783	68461783	57180064	**379.41**
Brains_2_Blanco	14489590	14489590	12041090	**80.30**
Brains_3_Blanco	67738004	67738004	56525008	**375.39**
Brains_1_Rojo	25085265	25085265	20679418	**139.02**
Brains_2_Rojo	55130044	55130044	46079118	**305.52**
Brains_3_Rojo	34382456	34382456	28614778	**190.54**

**Calculated as (#reads*length)/genome_length*
